# Insights into the Mechanisms Underlying Boron Homeostasis in Plants

**DOI:** 10.3389/fpls.2017.01951

**Published:** 2017-11-17

**Authors:** Akira Yoshinari, Junpei Takano

**Affiliations:** Graduate School of Life and Environmental Sciences, Osaka Prefecture University, Sakai, Japan

**Keywords:** boron, channel, transporter, NIP, BOR, endocytosis, polar localization

## Abstract

Boron is an essential element for plants but is toxic in excess. Therefore, plants must adapt to both limiting and excess boron conditions for normal growth. Boron transport in plants is primarily based on three transport mechanisms across the plasma membrane: passive diffusion of boric acid, facilitated diffusion of boric acid via channels, and export of borate anion via transporters. Under boron -limiting conditions, boric acid channels and borate exporters function in the uptake and translocation of boron to support growth of various plant species. In *Arabidopsis thaliana*, NIP5;1 and BOR1 are located in the plasma membrane and polarized toward soil and stele, respectively, in various root cells, for efficient transport of boron from the soil to the stele. Importantly, sufficient levels of boron induce downregulation of NIP5;1 and BOR1 through mRNA degradation and proteolysis through endocytosis, respectively. In addition, borate exporters, such as *Arabidopsis* BOR4 and barley Bot1, function in boron exclusion from tissues and cells under conditions of excess boron. Thus, plants actively regulate intracellular localization and abundance of transport proteins to maintain boron homeostasis. In this review, the physiological roles and regulatory mechanisms of intracellular localization and abundance of boron transport proteins are discussed.

## Boron Nutrition and Toxicity in Plants

Boron (B) is an essential micronutrient for plant growth. The available form of B for plants is boric acid. Boric acid is a weak Lewis acid which forms borate anion: $B{\left(OH\right)}_{3}+H_{2}O\overset{\leftarrow }{\to }B{{\left(OH\right)}_{4}}^{-}+H^{+}$ (p*K*a = 9.24). Boric acid is relatively soluble and easily leached by rainfall. Therefore, B deficiency often occurs in high rainfall areas such as Southeast Asia and Southeast China (Shorrocks, 1997). In plant cells, borate covalently crosslinks two chains of pectin at rhamnogalacturonan II (RG-II) regions to form a network in the cell wall (Funakawa and Miwa, 2015). Pectin is an abundant polysaccharide in the primary cell wall and important in determining cell size and shape in higher plants. The requirement for B in plant species correlates well with pectin content (Hu et al., 1997). Functions of B in the cytoskeleton and membrane have also been suggested (Bassil et al., 2004; Voxeur and Fry, 2014).

Boron is toxic when present in excess. Excessive B accumulation is mostly found in arid and semi-arid areas such as South Australia and the Middle East (Nable et al., 1997). B toxicity affects various aspects of cellular metabolism, causes DNA damage, and frequently results in tissue necrosis (Reid et al., 2004; Sakamoto et al., 2011). To avoid B deficiency and toxicity, plants require B transport systems regulated by B conditions.

## Boron Transport Mechanisms

**FIGURE 1:**
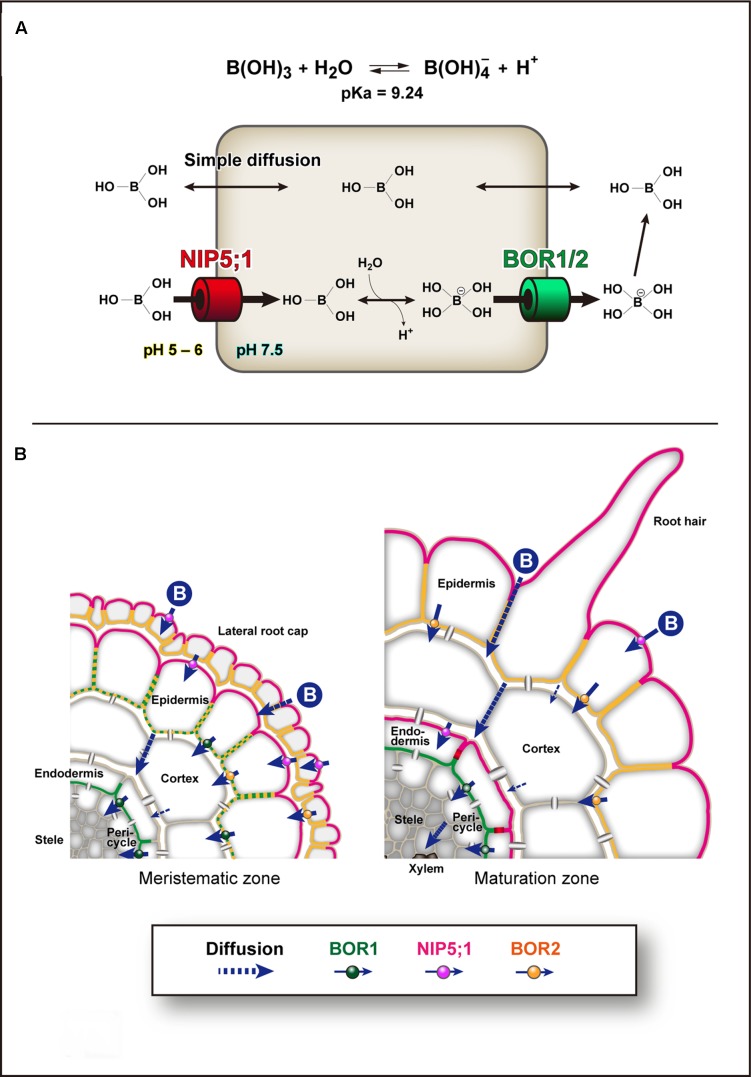
Models of Boron transport pathways. **(A)** Boron (B) transport across the plasma membrane. Boron transport and homeostasis are primarily based on three transport mechanisms across the plasma membrane (PM). The first is simple diffusion of uncharged boric acid across lipid bilayers. The second is facilitated diffusion of boric acid via NIP II boric acid channels, e.g., *Arabidopsis* NIP5;1. The third is export of borate via borate transporters, e.g., *Arabidopsis* BOR1 and BOR2. The boric acid channels transport boric acid into cells under physiological conditions. In the cytoplasm, which has a relatively high pH, boric acid is dissociated to borate anion and exported by borate uniporters driven by the electrochemical gradient. In the apoplast, which has a low pH, borate anion is rapidly converted to boric acid, and thus BORs can generate an uphill gradient of boric acid. **(B)** Cell-type specific expression patterns of NIP5;1, BOR1, and BOR2 in *Arabidopsis* roots under low-B conditions. NIP5;1 is expressed in lateral root cap and epidermis in the meristematic zone and epidermis and endodermis in the maturation zone (Wang et al., 2017). BOR1 is expressed in epidermis in meristem and endodermis in both meristematic and maturation zones (Yoshinari et al., 2016). BOR2 is expressed in lateral root cap and epidermis in both meristematic and maturation zones (Miwa et al., 2013). NIP5;1 is polarly localized in the soil-side PM domain (Takano et al., 2010). BOR1 and BOR2 are polarly localized in the stele-side PM domain (Takano et al., 2010; Miwa et al., 2013). The PM of endodermal cells is separated by the Casparian strip domain (Alassimone et al., 2010), and NIP5;1 and BOR1 are strictly separated to the two domains. Boric acid/borate is transported transcellulary from the soil to the xylem. In mature portions of roots, BOR1 at the stele side of endodermal cells is responsible for keeping boric acid/borate in the apoplasm in the stele.

Plant roots take up B as boric acid. Boric acid is a small, uncharged molecule, and is relatively permeable across biological membranes (Dordas and Brown, 2000; Stangoulis et al., 2001). Therefore, the passive diffusion of boric acid is considered to satisfy the plant demand for B when available in sufficient quantities. However, when the availability of boric acid is limited, plants use boric acid channels of the major intrinsic protein (MIP) family and the BOR family of borate exporters for transport of B (Takano et al., 2008). In addition, plants use BOR borate exporters for B exclusion from tissues under excess B conditions (Miwa et al., 2007; Sutton et al., 2007; Schnurbusch et al., 2010). The BOR family has a similar structure to anion transporters (Thurtle-Schmidt and Stroud, 2016; Coudray et al., 2017). A human BOR-like transporter, BTR1/SLC4A11, was characterized as an electrogenic, voltage-regulated Na^+^-coupled B(OH)_4_^-^ cotransporter by electrophysiology in human embryonic kidney (HEK) 293 cells (Park et al., 2004). HvBot1, a BOR homolog in barley that is involved in excess B tolerance, is a uniporter with high affinity for borate anion in *Xenopus* oocytes and patch-clamped proteoliposomes (Nagarajan et al., 2016). Therefore, BOR homologs likely function as borate anion uniporters driven by the negative membrane potential of plant cells. B transport and homeostasis are primarily based on three mechanisms of transport across the PM: passive diffusion of boric acid across lipid bilayers, facilitated diffusion of boric acid via boric acid channels, and export of borate, which is formed from boric acid in the cytoplasm, via BOR borate transporters (**Figure 1A**). Because of the low pH in the apoplast, borate anion is rapidly converted to uncharged boric acid, and thus BORs generate an uphill gradient of boric acid (+ borate).

To support plant growth and development, B must be preferentially transported to rapidly growing tissues when the availability of B is limited. The phloem mobility of B is highly divergent among plant species. In sugar alcohol-producing species, borate can bind to sugar alcohols such as mannitol, sorbitol, and dulcitol; the resulting complexes play a role in efficient B remobilization from old to young leaves through the phloem (Brown and Hu, 1996; Brown and Shelp, 1997; Hu et al., 1997). In sucrose-producing species, *bis*-sucrose borate and *bis-N-*acetyl-serine borate complexes were identified in wheat and canola phloem exudates, which explained the moderate phloem mobility of B (Stangoulis et al., 2010). The presence of these complexes is thought to be required to reduce leakage from phloem because boric acid can cross the PM.

## Functions and Regulation of Boric Acid Channels

Boric acid channels are aquaporin homologs belonging to the MIP family. Members of this family transport water and/or small, uncharged molecules. In plants, various MIPs transport boric acid and other small, uncharged molecules with different substrate specificities (Reid, 2014; Bienert and Bienert, 2017). MIPs are divided into PM intrinsic proteins (PIPs), nodulin 26-like intrinsic proteins (NIPs), small basic intrinsic proteins (SIPs), tonoplast intrinsic proteins (TIPs), and X intrinsic proteins (XIPs). NIPs are further divided into subclasses I, II, and III, depending on the channel pore structure (Roberts and Routray, 2017). The physiological role of NIP I proteins is not well-understood. NIP III proteins have been characterized as silicic acid channels (Ma and Yamaji, 2006). Here, we focus on NIP IIs, which are physiologically significant in B transport.

*Arabidopsis* NIP5;1 was the first identified boric acid channel (Takano et al., 2006). NIP5;1 loss-of-function mutants show severe growth reduction accompanied with low B uptake into roots under B-limiting conditions. NIP5;1 is expressed in the rhizodermis (outermost cell layers in roots) in the root tip and the endodermis in the mature root under B-limiting conditions (Takano et al., 2010; Wang et al., 2017). In the PM of these cell types, NIP5;1 shows polar localization toward the soil side. The function of NIP5;1 in B uptake is required only under B limitation (Takano et al., 2006) and the *NIP5;1* mRNA level is controlled in response to cellular B concentration by post-transcriptional regulation (Tanaka et al., 2011). This regulation depends on the minimum upstream open reading frame (uORF), *AUGUAA*, in the 5′-untranslated region (5′ UTR) of the mRNA (Tanaka et al., 2016). Higher B conditions enhance ribosome stalling at the AUG-stop and lead to suppression of translation and mRNA degradation. This downregulation is important for acclimation of plants to excess B conditions (Tanaka et al., 2011).

AtNIP6;1 is the closest paralog to AtNIP5;1. In NIP6;1 loss-of-function mutants, expansion of young leaves and apical dominance are disturbed under B-limiting conditions (Tanaka et al., 2008). NIP6;1 is expressed in phloem companion cells, parenchyma cells, and sieve elements in nodes, and is involved in B transfer from xylem to phloem (Tanaka et al., 2008).

AtNIP7;1 was first reported as an arsenite transporter (Isayenkov and Maathuis, 2008), and then characterized as an anther-specific boric acid channel (Li et al., 2011). NIP7;1 is expressed only in developing pollen microspores. Interestingly, the B transport activity of NIP7;1 is lower than that of NIP5;1 and NIP6;1 in *Xenopus* oocytes. However, substitution of Tyr-81 in the transport pore to Cys conferred on NIP7;1 higher B transport activity than that of NIP5;1 or NIP6;1. The authors proposed that the pore size of NIP7;1 might be controlled by pH, phosphorylation, and protein–protein interactions (Li et al., 2011). The physiological function of NIP7;1 in pollen remains to be studied.

In rice and maize, the NIP II proteins OsNIP3;1 and ZmTLS1 function as boric acid channels and are required for B transport under B-limiting conditions during vegetative and reproductive growth (Durbak et al., 2014; Hanaoka et al., 2014; Leonard et al., 2014). The conservation of NIP II in higher plants indicates the importance of boric acid channels for plant growth under B-limiting conditions.

## Physiological Functions of BOR Borate Exporters

The *Arabidopsis thaliana* genome harbors seven genes encoding BOR-type borate transporters (BOR1–BOR7) (Takano et al., 2002, 2008). In angiosperms, BORs can be divided into two distinct evolutionary clades (clades I and II) (Wakuta et al., 2015). Microarray analyses showed that BOR genes are expressed in various tissues during all developmental stages in *Arabidopsis* (Schmid et al., 2005; Winter et al., 2007).

AtBOR1, the prototype of clade I, was identified through analysis of the *bor1-1* (*requires high boron 1-1*) mutant (Noguchi et al., 1997; Takano et al., 2002). The loss-of-function mutant *bor1-1* showed severely reduced shoot growth under B-limiting conditions and sterility under normal-B conditions (Noguchi et al., 1997; Takano et al., 2001). These defects were associated with low-B accumulation in shoot tissues and were recovered by higher-B supply. In *Arabidopsis thaliana* seedlings, BOR1 is expressed in roots, hypocotyls, and cotyledons (Takano et al., 2010; Yoshinari et al., 2016). BOR1 is ubiquitously expressed in root tip cells and shows relatively strong expression in epidermal, endodermal, and provascular cells. In mature roots and hypocotyls, BOR1 is preferentially expressed in endodermal cells. In cotyledons, BOR1 is expressed in epidermal cells. Remarkably, BOR1 localizes to the PM in a polar manner toward the stele/vasculature in these cell types (Takano et al., 2010; Yoshinari et al., 2016; **Figure 1B**). This polar localization of BOR1 is likely important for efficient transport of B from the root surface to the xylem, in collaboration with NIP5;1. Although localization in the shoot is unclear, AtBOR1 is apparently involved in preferential B translocation at nodes and/or basal leaves in collaboration with NIP6;1 (Takano et al., 2001; Tanaka et al., 2008). AtBOR1 expressed in the epidermal cell layer of the cotyledon (Yoshinari et al., 2016) may be involved in B uptake at the surface of the cotyledon.

OsBOR1, ZmRTE, and BnaC4.BOR1;1c have been characterized as B transporters, and are required for B transport under B-limiting conditions *in planta* and are thus considered to be AtBOR1 orthologs in rice, maize, and rapeseed, respectively (Nakagawa et al., 2007; Chatterjee et al., 2014; Zhang et al., 2017). The expression and B export activity of BOR1 homologs in grapevine, citrus, and wheat have also been reported (Pérez-Castro et al., 2012; Cañon et al., 2013; Leaungthitikanchana et al., 2013).

AtBOR2, which has 90% identity to AtBOR1, is distributed in the PM with stele-side polarity, similar to AtBOR1 (Miwa et al., 2013). In contrast to AtBOR1, AtBOR2 is preferentially expressed in the root cap and epidermal cells rather than the inner cell layers of the root tip (Miwa et al., 2013). In the roots of the *AtBOR2-*knockout mutants *bor2-1* and *bor2-2*,B–RG-II cross-linking rates and cell elongation were significantly reduced compared to the *AtBOR1*-knockout mutant *bor1-3* under B-limiting conditions (Miwa et al., 2013). Therefore, BOR2 promotes B–RG-II cross-linking in root cells to support root growth. Pectin chain is assumed to be synthesized in the Golgi and Golgi-derived vesicles (Harholt et al., 2010). An analysis of RG-II in *Rosa* cells cultured in B-free medium showed that re-addition of boric acid resulted in gradual appearance of the RG-II dimer without detectable loss of existing monomers in the cell wall (Chormova et al., 2014). Consistently, RG-II monomer in the cell wall was not cross-linked when *de novo* biosynthesis of polysaccharides was pharmacologically inhibited. These results suggest that only newly synthesized RG-II was cross-linked by borate during or just after secretion from the Golgi to the cell wall. Interestingly, BOR2–GFP was not stably localized in the PM but showed cycling between the endomembrane compartments and the PM (Miwa et al., 2013). It is possible that BOR2 functions to transport B into secretory vesicles to promote cross-linking of RG-II.

AtBOR4 belongs to clade II and is involved in excess B tolerance. BOR4 is expressed in the epidermal and columella cells in the root tip and endodermal cells in the mature portions of the root (Miwa et al., 2007, 2014). Excess B conditions enhance accumulation of *BOR4* mRNA (Miwa et al., 2014). This upregulation was shown to be dependent on a heme oxygenase 1 (HO1) via its catalytic by-products (Lv et al., 2017). Unlike BOR1/2, BOR4 shows weak polar localization toward the soil side in root epidermal cells (Miwa et al., 2007; Łangowski et al., 2010). Under excess B conditions, *BOR4* T-DNA insertion lines accumulated more B in the shoots and roots and showed a greater reduction in growth than wild-type plants (Miwa et al., 2014; Lv et al., 2017). Therefore, BOR4 functions primarily in B exclusion from tissues. In barley and wheat, HvBot1 and its homologs are considered orthologs of AtBOR4 and have been identified as key factors for excess B tolerance (Sutton et al., 2007; Pallota et al., 2014; Reid, 2014).

In rice, OsBOR4, which belongs to clade II, is specifically expressed in pre-anthesis anthers and mature pollen (Tanaka et al., 2013). Disruption of *OsBOR4* disturbed pollen germination and elongation, suggesting that OsBOR4 maintains intracellular B levels in pollen. Microarray data indicate that *AtBOR4* mRNA accumulation level is highest in the stamen (Schmid et al., 2005). AtBOR4 may function in B homeostasis for fertilization in addition to the exclusion of B for excess B tolerance.

Genes encoding BORs have been identified in bryophytes, non-vascular plants, and lycophytes, the most primitive extant vascular plants (Wakuta et al., 2015). BORs in the bryophyte *Physcomitrella patens* were classified differently from clades I and II, while BORs in the lycophyte *Selaginella moellendorffii* were classified as clades I and II. The B transport function of BORs in the bryophyte *Physcomitrella patens* is unclear. However, SmBOR1 (clade I) and SmBOR3 and 4 (clade II) function as B exporters when expressed in yeast cells (Wakuta et al., 2015). Although the physiological significance of these genes has not been elucidated, the common ancestor of vascular plants had likely already acquired two types of BOR for limited and excess B tolerance.

## Polar Localization of AtNIP5;1

In root cells of *Arabidopsis*, NIP5;1 and BOR1 show polar localization toward the soil and stele sides, respectively (Takano et al., 2010, 2017; Yoshinari et al., 2016; Wang et al., 2017; **Figure 1B**). Increasing numbers of nutrient transporters and aquaporins show polar localization in plant cells (Barberon and Geldner, 2014; Naramoto, 2017; Takano et al., 2017). Recently, a structure-localization analysis of NIP5;1 identified that a conserved ThrProGly (TPG) repeat in the N-terminal cytosolic region is required for polar localization (Wang et al., 2017). Phosphorylation of the Thr residues in the TPG repeat induces clathrin-mediated endocytosis and mediates the strong polar localization. These results indicate that continuous polar cycling between the PM and endosomal compartments is required for maintenance of polar localization against the lateral diffusion in the PM. Use of the NIP5;1 weak polar variant with Thr to Ala substitutions indicated that the polar localization significantly contributes to B transport from the soil to the shoots. This demonstrates the physiological importance of polar localization in directional nutrient transport. We assume that the uphill gradient of boric acid (+ borate anion) from the cytosol to the apoplast generated by BOR1 (**Figure 1A**) can be canceled if NIP5;1 transports boric acid at the stele side of the cells.

## Polar Localization and Vacuolar Trafficking of AtBOR1

**FIGURE 2:**
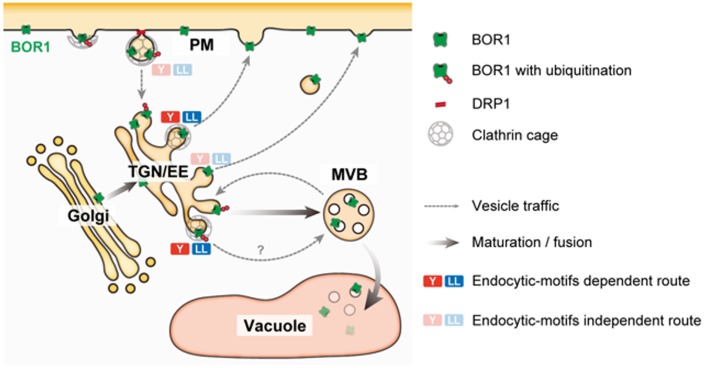
A model of post-Golgi trafficking of BOR1. BOR1 is internalized through clathrin and DRP1-dependent endocytosis and transported to the trans-Golgi network/early endosome (TGN/EE). Ubiquitinated BOR1 is sorted to intraluminal vesicles of multi-vesicular bodies (MVBs) by the endosomal sorting complexes required for transport (ESCRT) machinery. MVBs containing BOR1 fuse with the vacuole, releasing intraluminal vesicles. In the vacuole, BOR1 is immediately degraded by proteases. Unubiquitinated BOR1 is recycled to the PM from the TGN/EE via a clathrin-dependent or -independent route. BOR1 has endocytic motifs; three putative tyrosine motifs and a dileucine motif in the cytosolic loop region (Takano et al., 2010; Wakuta et al., 2015). These endocytic motifs bind to adaptor protein complexes involved in clathrin-dependent vesicle formation at the PM and TGN/EE (Bonifacino and Traub, 2003). BOR1 variants lacking either motif did not show polar localization or B-induced vacuolar transport, although these variants were normally endocytosed under low B conditions (Takano et al., 2010; Wakuta et al., 2015). These results suggest that the endocytic motifs of BOR1, rather than endocytosis are involved in polar recycling and vacuolar sorting of B from the TGN.

Under B-limiting conditions, AtBOR1 and AtBOR2 are localized to the PM in a polar manner toward the stele, but are rapidly transported to the vacuole for degradation upon B supply to sufficient levels (Takano et al., 2005; Yoshinari et al., 2012; Miwa et al., 2013). In contrast, AtBOR4 shows weak polar localization toward the soil side and stably accumulates under excess B conditions (Miwa et al., 2007). Downregulation of AtBOR1 and AtBOR2 is considered to be important to prevent over accumulation of B in shoots. Upon sufficient B supply, BOR1 is ubiquitinated at Lys-590 in the C-terminal tail domain (Kasai et al., 2011). Ubiquitinated BOR1 is transferred to the multi-vesicular body/late endosome (MVB/LE) and then to the vacuole for degradation (Viotti et al., 2010; **Figure 2**).

To understand BOR1 trafficking, single molecules of BOR1 in the PM were observed by variable-angle epifluorescence microscopy (VAEM; Yoshinari et al., 2016). With this technique, BOR1-GFP was visualized as particles in the PM and exhibited significant lateral movement in restricted areas. This is consistent with the relatively slow recovery of BOR1-GFP and other membrane proteins in the PM after photobleaching (FRAP analysis; Takano et al., 2010; Luu et al., 2012; Wang et al., 2017). The limited diffusion may contribute to maintenance of the polar localization of membrane proteins by vesicle trafficking. A portion of BOR1–GFP particles co-localized with DYNAMIN-RELATED PROTEIN 1A (DRP1A), which is involved in scission of clathrin-coated vesicles in the PM. They showed colocalization for 10–20 s, and then disappeared from the cell surface. Furthermore, a dominant-negative variant of DRP1A blocked endocytosis of BOR1 and disturbed its polar localization and vacuolar trafficking.

Polar localization toward stele and B-induced degradation are apparently important for the physiological roles of BOR1 under B-limiting conditions. The successful generation of a low B tolerant transgenic *Arabidopsis* by a *pro35S:AtBOR1* construct (Miwa et al., 2007) is considered to be dependent on BOR1 localization and regulation in various cell types. Interestingly, ubiquitous expression of a weakly polar and stabilized variant of AtBOR1 by introduction of a *proUBQ10:BOR1(K590A)-GFP-HPT* construct conferred excess B tolerance on *Arabidopsis* (Wakuta et al., 2016). This is similar to the case of overexpression of AtBOR4 in *Arabidopsis* (Miwa et al., 2007; Miwa and Fujiwara, 2011) and suggests that both evolutionary and artificial changes in the intracellular localization of transporters have differential physiological roles.

## Concluding Remarks and Future Perspectives

Characterization of members of the BOR and NIP II families has greatly advanced our understanding of B transport systems. To proceed, precise localization of transport proteins and measurement of B concentrations at the cellular level are required. This would be facilitated by laser ablation-inductivity coupled plasma-mass spectrometry (Iwai et al., 2006; Shimotohno et al., 2015) and development of genetically encoded or chemical sensors for boric acid. Our understanding of the regulatory mechanisms of B transport has also advanced. In particular, B-induced endocytosis and degradation of AtBOR1 (Takano et al., 2005, 2010; Kasai et al., 2011; Yoshinari et al., 2016) and B-induced ribosome stalling and mRNA degradation of AtNIP5;1 (Tanaka et al., 2011, 2016) are pioneering examples in plant nutrition. The next questions are the mechanisms underlying the B sensing that induces these responses. Using B transport proteins, limited and excess B tolerant transgenic plants have been generated (Miwa et al., 2006, 2007; Kato et al., 2009; Pang et al., 2010; Takada et al., 2014; Uraguchi et al., 2014; Mosa et al., 2016; Wakuta et al., 2016). The next step is to improve and apply these techniques to crop plants to enhance agriculture in areas of B deficiency and accumulation.

## Author Contributions

All authors listed have made a substantial, direct and intellectual contribution to the work, and approved it for publication.

## Conflict of Interest Statement

The authors declare that the research was conducted in the absence of any commercial or financial relationships that could be construed as a potential conflict of interest.
